# Exploring the human microbiome – A step forward for precision medicine in breast cancer

**DOI:** 10.1002/cnr2.1877

**Published:** 2023-08-04

**Authors:** Asmita Jotshi, Krishna Kishore Sukla, Mohammed Monzoorul Haque, Chandrani Bose, Binuja Varma, C. B. Koppiker, Sneha Joshi, Rupa Mishra

**Affiliations:** ^1^ Centre for Translational Cancer Research: A Joint Initiative of Indian Institute of Science Education and Research (IISER) Pune and Prashanti Cancer Care Mission (PCCM) Pune India; ^2^ Life Sciences R&D, TCS Research, Tata Consultancy Services Limited Pune India; ^3^ TCS Genomics Lab, Tata Consultancy Services Limited New Delhi India; ^4^ Prashanti Cancer Care Mission, Pune, India and Orchids Breast Health Centre, a PCCM initiative Pune India

**Keywords:** AI/ML, biomarker, breast cancer, microbiome, oncobiome, precision oncology

## Abstract

**Background:**

The second most frequent cancer in the world and the most common malignancy in women is breast cancer. Breast cancer is a significant health concern in India with a high mortality‐to‐incidence ratio and presentation at a younger age.

**Recent Findings:**

Recent studies have identified gut microbiota as a significant factor that can have an influence on the development, treatment, and prognosis of breast cancer. This review article aims to describe the influence of microbial dysbiosis on breast cancer occurrence and the possible interactions between oncobiome and specific breast cancer molecular subtypes. The review further also discusses the role of epigenetics and diet/nutrition in the regulation of the gut and breast microbiome and its association with breast cancer prevention, therapy, and recurrence. Additionally, the recent technological advances in microbiome research, including next‐generation sequencing (NGS) technologies, genome sequencing, single‐cell sequencing, and microbial metabolomics along with recent advances in artificial intelligence (AI) have also been reviewed. This is an attempt to present a comprehensive status of the microbiome as a key cancer biomarker.

**Conclusion:**

We believe that correlating microbiome and carcinogenesis is important as it can provide insights into the mechanisms by which microbial dysbiosis can influence cancer development and progression, leading to the potential use of the microbiome as a tool for prognostication and personalized therapy.

## 1 INTRODUCTION

Breast cancer is a complex and multifactorial disease that poses a significant global health concern, According to the World Health Organization (WHO), it is the most common cancer among women worldwide, with an estimated 2 million new cases and a high incidence to mortality ratio.^1^, ^2^ In India, 27% of all cancer cases are Breast Cancer.^3^ These numbers highlight the urgent need for effective diagnostic and prognostic tools to improve breast cancer management. In terms of breast cancer management, global efforts focus on early detection, diagnosis, and treatment. Screening programs help detect breast cancer at an early stage when it is more treatable. However, breast cancer management and its manifestation differ in India compared to global trends. In India, breast cancer often presents at advanced stages due to factors like lack of awareness, limited access to healthcare, and cultural barriers. Additionally, breast cancer in India affects women at a younger age compared to Western countries.^4^ This early onset may be influenced by genetic factors, reproductive patterns, and hormonal influences. The exploration of the microbiome as a diagnostic and prognostic tool holds great potential in the management of breast cancer, particularly in the Indian context.

The human microbiome, composed of trillions of microorganisms, plays a significant role in health and disease. The majority of diverse microbial species are found in the distal gastrointestinal tract^5^, ^6^ so the intestinal microbiota plays a vital role in many physiological processes. Various studies have explored the composition of the normal flora of the gut. Although a consensus healthy, gut microbiota cannot be defined since, gut microbial composition is specific to individuals, certain characteristics that correlate with healthy gut microbiota, such as high taxonomic diversity and microbial gene richness, have been described.^7^, ^8^, ^9^

Dysbiosis, characterized by an imbalance in the microbial community, has been associated with various diseases, such as metabolic syndrome, inflammatory bowel disease, and cancer.^10^ Studies suggest that microbial dysbiosis can contribute to carcinogenesis through mechanisms such as metabolite production, inflammation, immune dysregulation, DNA damage, and altered drug metabolism.^11^, ^12^, ^13^, ^14^, ^15^, ^16^, ^17^ Human microbiome research has emerged as a promising field with transformative potential in the diagnosis, monitoring, treatment, and management of various diseases, including cancer. The WHO's World Cancer Report–2020 highlights the role of the human microbiota in cancer development and therapy.^18^ Recent years have witnessed a growing interest in understanding the interplay between the human microbiome and tumorigenesis, particularly the intricate relationship termed “cancer‐microbiome crosstalk”.^19^ In addition to its role in cancer development, the gut microbiota has been found to influence the response to different cancer treatments, including chemotherapy, radiation therapy, and immune‐based therapies.^20^ With respect to breast cancer, dysbiosis not just in gut microbiota but also the breast tissue microbiota has been reported.^21^ The interaction between the human microbiome and cancer has been recently coined as “Oncobiome”. Understanding this “cancer‐microbiome crosstalk” and exploring the associations between microbiome composition and various breast cancer subtypes could offer insights into the mechanisms underlying breast cancer and may pave the way for novel diagnostic and treatment strategies. Moreover, studying the crosstalk between the gut microbiome and breast tissue microbiome provides a deeper understanding of how these microbial communities contribute to the heterogeneity of breast cancer and influence its clinical behavior.^22^

In this review, we have attempted to provide an overview on the potential role of the human microbiome in tumorigenesis, specifically in relation to breast cancer. The crosstalk between the microbiome and breast cancer, and the influence of this interplay on epigenetics and nutrition, has been explored, shedding light on potential mechanisms and avenues for targeted interventions. Additionally, the review discusses perspectives on emerging technologies, such as Next Generation Sequencing, for microbiome sampling and analysis, both experimental and in silico. By synergizing the existing knowledge and exploring emerging perspectives, this review aims to provide a comprehensive understanding of the microbiome's role in breast cancer.

## 2 THE MICROBIOME IN CANCER

Various studies report the influence of the gut microbiome not just on the gut microenvironment but also its influence on the physiology of distant organs including the breast.^23^, ^24^, ^25^, ^26^ The gut microbiota in breast cancer patients is reported to differ from healthy individuals, with differences in alpha and beta diversity reported in breast cancer patients.^27^, ^28^, ^29^ The tumor microenvironment (TME) in various cancers, including lung, pancreatic, colon, and liver, has a distinct microbiome that influences cancer development and response to therapy. The airway microbiota drive the chronic inflammation required for lung adenocarcinoma development through the activation of IL‐17‐producing *T* cells. In the pancreatic ductal adenocarcinoma (PDAC) model, the intratumoral microbiome creates an immunosuppressive TME by activating toll‐like receptors on monocytic cells, promoting tumor growth. Similarly, in a genetically induced model of colon cancer, gut microbiota induce IL‐6 secretion, leading to the inhibition of antitumor CD8+ *T* cells. Germ‐free mice implanted with lymphoma tumors demonstrated the tumor‐promoting potential of the microbiome, as did the specific microbiome composition that increased colon tumorigenesis.^30^, ^31^, ^32^ The gut microbiome also plays a role in liver tumor development and metastasis through the conversion of primary bile acids to secondary bile acids. However, indole compounds, resulting from tryptophan metabolism by gut bacteria, promote pancreatic cancer development by inducing inflammatory polarization of tumor‐associated macrophages. These findings highlight the crucial role of the microbiome in cancer development and its potential as a therapeutic target.

## 3 MICROBIOME AND BREAST CANCER

Understanding the oncobiome specific to breast cancer is crucial for effective personalized treatment options. As studies have shown, significant differences exist in the microbial signatures of breast cancer patients compared to healthy individuals, and these differences appear to vary based on the molecular subtype. Breast cancer is classified into three major subtypes based on the expression of certain cell surface receptors. The Estrogen‐receptor and/or progesterone receptor expressing cells are classified as ER/PR positive breast cancer, Human Epidermal Growth Factor Receptor 2 (HER2) positive or the subtype that does not express any of these receptors, termed Triple Negative Breast Cancer. The unique microbial signatures identified among the subtypes in a study by Banerjee et. al. suggests subtype‐specific oncobiome alterations in breast cancer. According to the study, women with breast cancer had higher relative abundances of *Bacillus*, *Enterobacteriaceae*, and *Staphylococcus* in their breast tissue compared to healthy controls. Specifically, *Escherichia coli* (a member of the *Enterobacteriaceae* family) and *Staphylococcus epidermidis*, isolated from breast cancer patients, were shown to induce DNA double‐stranded breaks in HeLa cells using the histone‐2AX (H2AX) phosphorylation (γ‐H2AX) assay. The study also found a decrease in some lactic acid bacteria, known for their beneficial health effects, including anticarcinogenic properties, in breast cancer patients^33^

Estrogen receptor‐positive breast cancer (ER+) is a type of breast cancer fueled by the hormone estrogen. The estrobolome, the collection of bacterial genes responsible for metabolizing estrogens, is thought to play a significant role in regulating estrogen levels in the body by affecting the enterohepatic circulation of estrogens. Bacterial species possessing β‐glucuronidases and β‐glucuronides are particularly important in estrogen metabolism, as they are involved in estrogen deconjugation and conjugation. This reactivation allows unbound estrogens to be recirculated through the bloodstream, possibly influencing hormonal disorders, including ER/PR+ breast cancer.^34^, ^35^, ^36^, ^37^, ^38^ Studies have shown that certain bacteria in the gut microbiome can increase the levels of estrogen in the body, thus promoting the growth and spread of ER+ breast cancer cells. Microbial dysbiosis can lead to the production of harmful metabolites and inflammatory molecules, which can contribute to the development and progression of breast cancer by modulating host immune responses and inflammatory pathways, favoring tumorigenesis and progression.^39^, ^40^ Moreover, studies suggest that the gut microbiome could influence the effectiveness of estrogen‐targeted therapies for breast cancer. While the specific positive or negative influence of the gut microbiota on the hormonal treatment of breast cancer remains unknown, some studies show, *Bacteroides fragilis* was specifically found in young women of premenopausal statuses and *Klebsiella pneumonia* in older women of postmenopausal statuses. These microbial markers were identified in different menopausal statuses of breast cancer, and their presence exhibited excellent discriminatory ability in distinguishing breast cancer patients from controls.^33^, ^41^, ^42^ Other studies using shotgun metagenomic analysis, show at least 45 bacterial species showed significant differential abundance between postmenopausal breast cancer patients and controls, including *Fusobacterium varium, Shigella_sp_D9, Desulfovibrio piger*, *Escherichia_sp_1_1_43*, *Shigella sonnei*, *Eubacterium eligens*, *Escherichia_sp_3_2_53FAA*, *Vibrio cholerae*, *Acinetobacter baumannii*, *Proteus mirabilis*, *Fusobacterium nucleatum*, *Campylobacter concisus*, *Escherichia coli*, and *Porphyromonas uenonis*. Furthermore, gene annotation showed a higher abundance of pathogen‐host interaction genes as well as virulence factors in postmenopausal women with breast cancer compared to controls. Lastly, there was a difference in functional pathways between postmenopausal women with breast cancer and controls, including the enrichment of lipopolysaccharide biosynthesis, iron complex transport system, vitamin B12 transport system, PTS system, secretion system, and beta‐oxidation genes, while butyrate synthesis was diminished in postmenopausal women with breast cancer.^29^ These findings suggest that understanding the role of the gut microbiome in the development and progression of ER‐positive breast cancer and the efficacy of estrogen‐targeted therapies may lead to better prevention and treatment strategies for breast cancer.

HER2+ breast cancer is also reported to have distinct microbial signatures compared to HER2‐ breast cancer. Studies examining gut and tissue microbiota have shown a prevalence of *Proteobacteria*, *Firmicutes*, and *Bacteroidetes* in HER2+ breast cancer.^43^, ^44^, ^45^, ^46^ While the mechanistic correlation of microbiota in predisposition to HER2+ breast cancer is yet to be delineated, recent reports suggest that microbiota can affect treatment response to HER2 targeted therapy. Trastuzumab, a HER2 targeted therapy, prevents HER2 receptor dimerization and activates the innate immune response.^47^ However, differences in gut microbiota brought about by usage of antibiotics impact the activation of the innate immune response, thus affecting the efficacy of trastuzumab therapy.^27^, ^48^ Studies have thus demonstrated the active role of microbiota in trastuzumab response, and modulation of this microbiome may facilitate better treatment response. However, an understanding of the mechanism is essential before such therapeutic approaches can be implemented.

Triple‐negative breast cancer (TNBC) is an aggressive subtype of breast cancer associated with a poor prognosis, and there are currently no targeted therapies available. Moreover, in India, the proportion of TNBC cases is higher compared to the western population^49^, ^50^ and microbiome analysis may provide insights into the reason for this disparity. Studies have found that the microbiome of TNBC tumors is distinct from that of adjacent normal breast tissue and contains higher levels of certain bacteria, such as *Fusobacterium* and *Prevotella*. These bacteria have been linked to inflammation and tumor growth in other types of cancer. Other studies have shown that TNBC has the least taxonomic diversity among all breast cancer types and a higher abundance of biofilm‐forming bacterial strains, such as *Aggregatibacter* and *Caulobacter*. The microbial profiles found in adjacent normal tissue were more similar to the tumor microbiome than those found in non‐matched healthy tissue controls, suggesting that the TME resident tumor microbiota can extend to surrounding tissues, implying an active role in tumorigenesis.^13^, ^43^, ^51^ Recent evidence also supports the idea that intra‐tumoral bacteria can localize within the tumor and immune cells in various cancer types, including TNBC. Intriguingly, recent studies suggest that the influence of gut microbiome on tumor‐infiltrating lymphocytes (TILs) profile is associated with better treatment outcomes in TNBCs and other cancers. Certain bacterial species for example, *Fusobacterium nucleatum*, and *Staphylococcus aureus* known to colonize TNBC tumors have been found to shift TILs populations and induce a tumor‐suppressing pro‐inflammatory (Type 1) immune responses^52^, ^53^

Moreover, patients with diversity in the microbiome population (eubiosis) show a better response to treatment and overall survival as compared to patients with reduced microbiome diversity (dysbiosis).^54^, ^55^, ^56^ This was confirmed in a mice study where one group received a fecal microbiota transplant (FMT) from responders and showed improvement in their response to anti‐PDL1, while those who received an FMT from non‐responders developed resistance.^57^

While the emerging evidence suggests that TNBC is associated with distinct microbial signatures that may play a role in tumor growth, metastasis, and response to treatment, more research is needed to fully understand the relationship between the TNBC microbiome and tumor biology. Future studies may determine whether interventions aimed at modulating the microbiome can improve treatment outcomes for patients with this challenging breast cancer subtype. *Prevotella* and *Faecalibacterium* are reported to be enriched in the gut microbiome of the Indian population and have been categorized as comprising the core Indian gut microbiota.^58^, ^59^ Some strains of *Prevotella* have been associated with beneficial effects while some have been reported to have pathobiontic properties.^60^ Interestingly studies assessing the differential microbiota in TNBCs identified *Prevotella* as the dominant species in TNBC patients compared to healthy individuals in western population.^51^, ^61^ Further studies are needed to decipher the role of *Prevotella* species in breast cancer, especially TNBC risk in the Indian context. *Faecalibacterium prausnitzii* on the other hand is reported to have protective effects and inhibits the proliferative capacity of breast cancer cells through suppression of IL6 and via JAK2/STAT3 pathway.^62^ Certain studies have also shown an inverse correlation of *Faecalibacterium* with breast cancer.^63^, ^64^ Additionally, presence of *Faecalibacterium* seems to indicate better response to immunotherapy.^41^, ^65^

These contrasting reports suggest the need for population specific studies to determine the exact correlation of microbiome to breast cancer risk and treatment response. It remains to be determined whether the differential gut microbiome of the Indian population may be one of the factors for the higher proportion of TNBC cases. Additionally, immunotherapy due to many factors is not yet widely utilized in India. Given the abundance of *Faecalibacterium* in the gut microbiome of the Indian population, it may be probable that breast cancer patients in India may respond well to immunotherapeutic approaches. However, the interplay of these organisms with other commensals in the Indian population and their impact on breast cancer risk and treatment response remains to be elucidated.

## 4 POTENTIAL IMPLICATIONS OF THE MICROBIOME ON ONCO‐THERAPEUTICS 

We have previously discussed the differences in microbiome composition in the article. To summarize in brief, benign breast tissue samples have greater mean relative abundances of 11 genera compared to cancer‐associated samples. On the other hand, higher relative abundances of bacteria that had the ability to cause DNA damage in vitro were found in breast cancer patients.^45^ Interestingly, some lactic acid bacteria known for their beneficial health effects, including anticarcinogenic properties, were decreased in breast cancer patients. *Prevotella, Lactococcus, Streptococcus*, *Corynebacterium*, and *Micrococcus* were significantly more abundant in healthy patients, while *Bacillus*, *Staphylococcus*, *Enterobacteriaceae* (unclassified), *Comamondaceae* (unclassified), and *Bacteroidetes* (unclassified) were more abundant in cancer patients.^33^ Postmenopausal women diagnosed with incident breast cancer showed significantly lower alpha diversity in fecal microbiota and differing relative abundance of select taxa of *Firmicutes*, with higher grade breast cancer being associated with higher abundance of *Actinobacteria* (*g_Eggerthella*) but lower abundance of other taxa of *Actinobacteria* (*f_Coriobacteriaceae*), and *Firmicutes* (*f_Lachnospiraceae*, *g_Anaerostipes*, *f_Ruminococcaceae*), and higher stage breast cancer being associated with higher abundance of *Firmicutes* (*f_Clostridiaceae*) and *Proteobacteria* (*f_Enterobacteriaceae*, *g_Haemophilus*) but lower abundance of Firmicutes (*g_Acidaminococcus*, *g_Catenbacterium)*.^28^ Specific microbes such as *Escherichia coli*, *Klebsiella sp_1_1_55*, *Prevotella amnii*, *Enterococcus gallinarum*, *Actinomyces* sp. *HPA0247*, *Shewanella putrefaciens*, and *Erwinia amylovora* have been enriched in postmenopausal breast cancer patients.^29^ The gut microbiome composition of breast cancer patients differs based on their estrogen receptor (ER) status, with patients with ER‐positive breast cancer having a higher abundance of *Lactobacillus* and *Bifidobacterium*, while patients with ER‐negative breast cancer had a higher abundance of *Fusobacterium*.^66^ Some studies have observed differences in the microbial composition of specific bacterial phylotypes that have been linked to breast cancer, such as *Proteobacteria*, *Actinobacteria*, *Firmicutes*, *Bacteroidetes*, *Sphingomonas yanoikuyae*, and *Methylobacterium radiotolerans* in breast tissue between individuals with breast cancer and those without the disease^42^, ^67^, ^68^, ^69^

Due to the gut microbiome's close interaction with the immune system, it has recently gained increased attention for its potential role in immunotherapy. The response rate of a patient to immunotherapy depends on immune competency, diversity, antigen specificity variation, antigen expression, and the recent identification of the role of gut microbiota. The microbiome plays a role in developing inflammation, the integrity of mucosal immunity, and protecting against pathogens.

Interactions between the gut microbiota and the host are highly complex and may explain the variable findings regarding the presence of certain bacterial species. For instance, the abundance of *Akkermansia muciniphila* was found to be higher in individuals with colorectal cancer in one study, but in another study, it was found to be a key factor in the effectiveness of anti‐PDL‐1 immunotherapy.^70^, ^71^, ^72^ These conflicting findings highlight the need for further research and suggest that the optimal abundance of key bacteria may be individualized based on the host, the disease, and the treatments. Therefore, it is crucial to consider how previous treatments and comorbid conditions may have impacted an individual's gut microbiota composition when planning future treatments, as dysbiosis may contribute to cancer pathogenesis and therapy. In a very recent study aimed to validate the prognostic significance of fecal *Akkermansia muciniphila* in advanced non‐small cell lung cancer (NSCLC) patients treated with first or second line Immune Checkpoint Inhibitors (ICI) showed Intestinal *Akkermansia muciniphila* predicts clinical response to PD‐1 blockade.^73^ Collectively many studies have shown that gut microbiome plays an important role in the ICI, with antibiotics negatively impacting ICI efficacy by altering the gut microbiome composition. Strategies need to be developed to improve the efficacy of immunotherapy that may include fecal microbiota transplantation, probiotics, prebiotics, dietary modifications, and antibiotic management.

The potential for precision medicine interventions based on the microbiome is promising, and further research in this area may lead to identifying new treatment avenues for breast cancer patients. Identification of the differential microbiome signature via fecal or blood samples from patients may help in diagnosis as well as predicting treatment response. Current avenues for precision medicine based on microbiome signature are thus focused on two major aspects. One is leveraging the understanding of the microbiome to predict treatment response and the other is to develop interventions aimed at modulating the microbiome composition to assist therapeutic strategies.

## 5 CROSSTALK BETWEEN TWO HALLMARKS OF CANCER ‐ THE MICROBIOME AND EPIGENETIC REGULATION 

Gut microbiota can have an impact on DNA methylation along with the regulation of DNA damage and repair pathways.^74^, ^75^ Although it has been reported that epigenetic regulation is one of the mechanisms by which microbiota can promote cancer, the association remains unclear in the case of breast cancer specifically. Certain phytochemicals in our diet can regulate the enzymes in epigenetic pathways thereby altering the expression of genes.^76^, ^77^ Dietary fibers that are metabolized by the gut microbiota produce monocarboxylic acid compounds as well as polyphenols. The monocarboxylic acid compounds produced include acetate, propionate, and butyrate collectively called the short‐chain fatty acid (SCFA) family, which can act as epigenetic regulators.^78^ For example, butyrate can inhibit the activity of histone deacetylases, thereby inducing tumor cell death by promoting apoptosis in breast cancer cell lines. Moreover, it can cause epigenetic modifications that activate tumor‐suppressor genes like p21. Epigallocatechin‐3‐gallate (EGCG) and isothiocyanate sulforaphane (SFN), two dietary polyphenols, can alter the composition of the gut microbiota and control epigenetic markers.^17^

Non‐protein coding miRNAs, one of the important components of epigenetic regulation, have also been studied for their association with gut microbiota. miRNAs are short, non‐coding RNAs that inhibit posttranscriptional gene expression and interfere with the translation process that controls metastasis, tumor cell survival, and other vital cancer‐related biological processes in breast cancer. Normal cells have tightly regulated miRNA expression, whereas cancer cells may have abnormal miRNA processing, which can promote tumorigenesis. Several circulating miRNAs that are indicative of and specific to breast cancer have been found, suggesting that these miRNAs may serve as possible biomarkers for diagnosing cancer.^79^ For example, miR21 is commonly used as a biomarker for recognition of metastasis levels, diagnosis, and prognosis. miR155 was investigated as a prognostic biomarker in early‐stage TNBC.^17^, ^80^ Few studies have reported that miRNAs in the tumor microenvironment may regulate the composition of the gut microbiome by controlling bacterial species, such as *Fusobacterium nucleatum* and *Escherichia col*i, which in turn affects gene regulation^81^, ^82^ However, more research is required to understand how epigenetics regulates the gut and breast microbiome and might thus contribute to breast cancer.

## 6 GUT HEALTH, DIET AND BREAST CANCER

Genetics, lifestyle, dietary habits, and physical activity are frequently discussed risk factors for breast cancer. While the genetic component is beyond external control, diet and lifestyle are modifiable risk factors. The gut microbiome, which influences various physiological processes including the fermentation of dietary fibers and the deactivation of toxins and carcinogens, plays a crucial role in breast cancer predisposition The diversity and composition of the gut microbiome are influenced by diet. A plant‐based diet, rich in fruits and vegetables containing bioactive components like polyphenols and fibers, promotes a diverse microbiome. On the other hand, a diet high in saturated fats, red meat, and sugars can lead to the production of harmful metabolic products by the microbiome, contributing to inflammation and cancer development.^56^ Body mass index (BMI) is also associated with differences in the microbiome. Overweight and obese women have been found to have a decreased number of certain beneficial *Firmicutes*, *Faecalibacterium prausnitzii* and *Blautia spp* compared to normal weight women.^83^ Obesity is linked to higher estrogen levels, which increase the risk of various cancers including breast cancer. Studies in mice have shown that a high‐fat diet can alter the gut microbiota and impact tumorigenesis.^84^

Macro and micronutrients such as fiber, vitamins, minerals, and phytonutrients are essential for maintaining a healthy gut microbiome. In cancer patients undergoing chemotherapy and immunotherapy, adequate nutrition plays a vital role in repopulating the gut microbiome and potentially influencing treatment response.^85^, ^86^, ^87^, ^88^, ^89^, ^90^

SCFAs from dietary fibers are known to promote the growth of beneficial bacteria, reduce inflammation, improve gut barrier function and may help protect against breast cancer.^91^ Polyphenols may protect against the development and progression of the breast cancer by reducing inflammation, regulating hormone levels, and promoting DNA repair.^92^ Studies have shown that polyphenols can increase the abundance of beneficial bacteria such as *Bifidobacterium* and *Lactobacillus*, while decreasing the abundance of harmful bacteria, such as *Clostridium perfringens* ^93^

Apart from this, several microbial strains can play a role in the synthesis of certain vitamins and cofactors.^94^ Several studies have found an association between vitamin A intake or plasma concentration of vitamin A and gut microbiota composition.^87^, ^95^ The potential protective role of vitamin A in breast cancer development is also under investigation, as Vitamin A is a powerful antioxidant that modulates oxidative stress and cancer development. Despite limited clinical application for the prevention and treatment of breast cancer, the epidemiological evidence supports Vitamin A association with reduced risk of breast cancer and potential therapeutic effects, as well as the impact of genetic variations involved in Vitamin A metabolism on cancer and other diseases^96^

There is a growing body of evidence to suggest that higher levels of vitamin D3 may be associated with a reduced risk of breast cancer. Some studies have found that vitamin D3 may have anti‐tumor effects and may also help regulate key cellular processes involved in breast cancer development and progression. Recent evidence also suggests a relationship between vitamin D3 and the microbiome in breast cancer development. Studies have shown that vitamin D3 can influence the gut microbiota and promote an anti‐inflammatory environment, potentially reducing breast cancer risk.^97^, ^98^ Additionally, the gut microbiota can affect vitamin D3 metabolism, and has been associated with increased breast cancer risk. A recent study examined the relationship between intestinal Vitamin D receptor (VDR) and breast cancer, specifically investigating whether an intestinal‐microbiome‐breast axis exists. The study found that VDR deficiency led to gut dysbiosis, increasing susceptibility to breast cancer induced by DMBA. Further, treatment with butyrate or *Lactobacillus plantarum* reduced breast tumors and inflammation, suggesting that gut‐tumor‐microbiome interactions may represent a new target in the prevention and treatment of breast cancer. The study provides insights into the potential role of the gut microbiome in the pathogenesis of breast cancer and highlights the importance of VDR in maintaining gut and breast health.^98^

**FIGURE 1:**
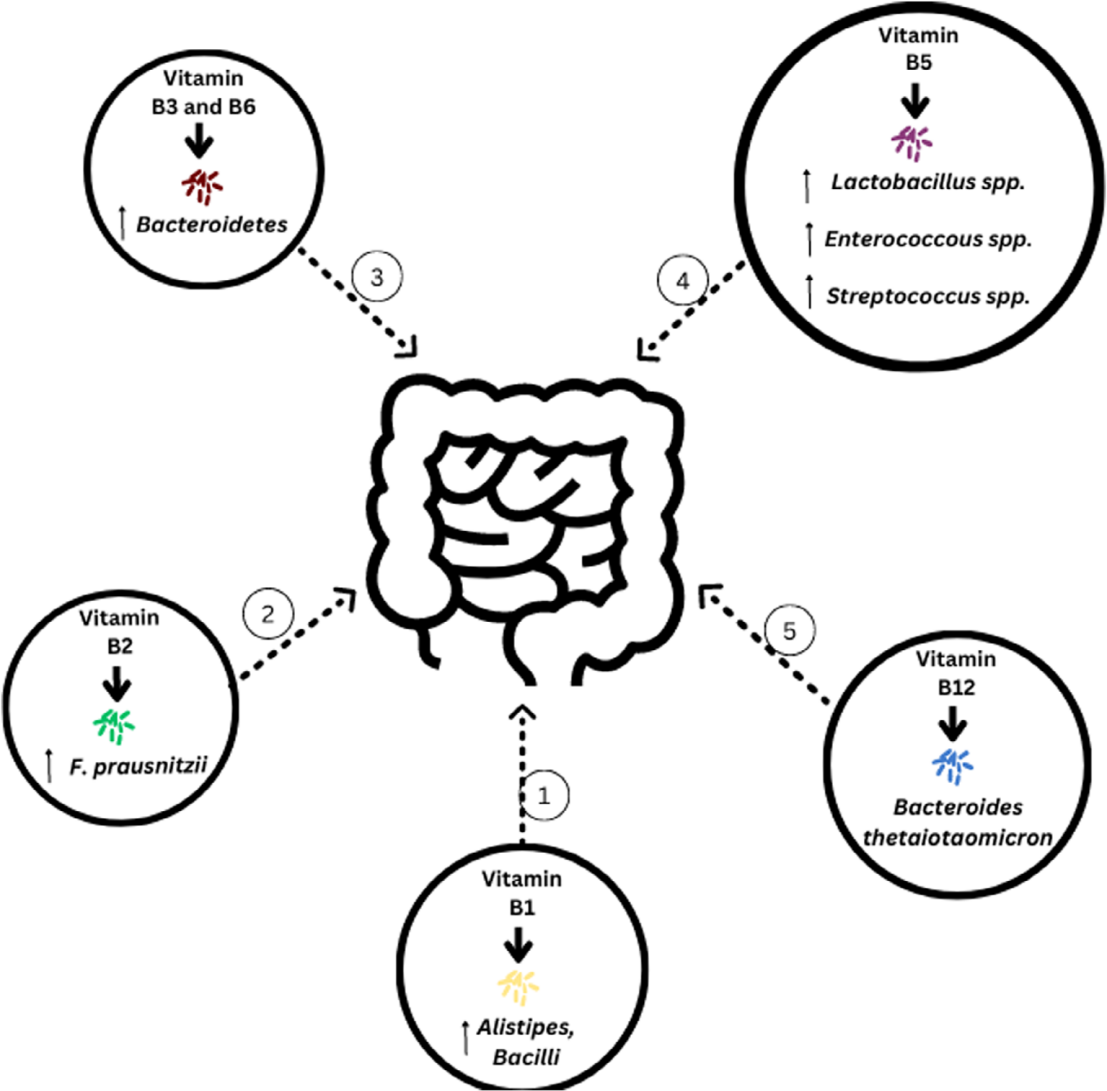
Schematic representation of effect of Vitamin B Complex on host gut microbiome 1. Members of genus *Alistipes* and *Bacilli* are said to be affected adversely by deficient levels of Vitamin B1^153^; 2. It has been observed that the growth of *F. prausnitzii* is stimulated by exposure to Vitamin B2^154^; 3. Vitamin B3 and B6 supplementation has been observed to stimulate increased abundance of *Bacteroidetes*, which is a part of the human gut commensal^155^; 4. The growth of Lactic Acid Bacteria like *Lactobacillus* spp., *Enterococcus* spp. and *Streptococcus* spp. is modulated by Vitamin B5 levels^155^; 5. Uptake of Vitamin B12 by *B. thetaiotaomicron*, which is a gut commensal, has shown to limit Shiga toxin production^155^; 6. In summary, dietary fibers, micronutrients, and phytonutrients are important for maintaining a healthy gut microbiome and may affect breast cancer risk. However, it is important to note that the interactions between dietary factors and its influence on gut microbiome and breast cancer are complex, and more research is needed to fully understand the role of these nutrients in reducing breast cancer risk.

On the other hand, a recent study has reported a positive correlation for Vitamin B1 with breast cancer although correlation of other B‐Vitamins with breast cancer risk is not yet established.^99^ Interestingly, B‐vitamins are known to be essential in determining the diversity and richness of gut flora, which is known to influence breast cancer risk^87^ and further studies are needed to help determine the interplay of B‐vitamins, the gut flora and breast cancer risk. Figure 1 shows the influence of various B‐vitamins on the gut microbiota.

Among the micronutrients, Selenium is reported to have a positive correlation with gut microbiome diversity and is vital for immune function.^100^, ^101^ Various studies are also investigating the association of zinc and magnesium with gut microbiome.^102^, ^103^, ^104^, ^105^ Magnesium is reported to be involved in many cellular processes and supports the growth of beneficial gut bacteria, while suppressing harmful bacteria like *Clostridium difficile*.^106^ Zinc is reported to improve gut barrier function and promote microbial diversity.^104^ Although Selenium and Magnesium are reported to be associated with improved survival^107^, ^108^, ^109^ the association of zinc with breast cancer risk is still ambiguous.^110^, ^111^ A study has reported that selenium may have a protective effect against breast cancer by regulating gene expression and reducing inflammation.^112^ Calcium also helps regulate bacterial growth and reduce inflammation, in addition to its role in maintaining healthy bones.^113^ Iron's effect on gut microbiome composition varies depending on the amount and form consumed.^114^ There is thus a need for further studies to delineate the exact correlation of these micronutrients with breast cancer risk and their interplay with the microbiome.

## 7 TECHNOLOGICAL ADVANCES IN BREAST/GUT MICROBIOME RESEARCH: A NEW FRONTIER FOR BREAST CANCER DIAGNOSTICS & THERAPEUTICS

Technological advances in gut microbiome research, including next‐generation sequencing (NGS) technologies, single‐cell sequencing, microbial metabolomics, meta‐transcriptomics, and microfluidics have the potential to revolutionize breast cancer diagnostics and therapeutics.^115^, ^116^ NGS technologies allow for rapid and comprehensive sequencing of the genetic material of the gut microbiome, revealing previously unknown microbial diversity and functional pathways.^117^, ^118^ This has led to the identification of specific microbial taxa and functional pathways associated with breast cancer development, such as changes in the abundance of certain bacteria or alterations in the production of specific metabolites.^119^ Similarly, genome sequencing and single‐cell sequencing enable researchers to identify specific genetic pathways and interactions between microbial species in the gut that may contribute to breast cancer development.^120^ By identifying these pathways and interactions, researchers may develop targeted therapies that modulate the gut microbiome to prevent or treat breast cancer.^83^ Microbial metabolomics provides new insights into the metabolic interactions between gut microbes and the host, which may be particularly relevant to breast cancer due to the role of metabolism in cancer development and progression. By measuring the small molecule metabolites produced by gut microbes, researchers may identify specific metabolic pathways associated with breast cancer and develop targeted therapies to modulate these pathways.^121^, ^122^ Hence, technological advances in gut microbiome research have the potential to significantly advance breast cancer diagnostics and therapeutics.

The recent advancements in artificial intelligence (AI) have opened new avenues for analyzing microbiome data.^123^, ^124^ Among the cutting‐edge techniques, deep neural networks and graph convolutional networks are used to identify complex patterns and interactions within microbial communities.^125^, ^126^ Bayesian networks can also be employed to explore the causal links between microbiome and health outcomes.^127^ The potential of these AI techniques is immense in unveiling critical insights into the role of the microbiome in human health and developing personalized treatments. Specifically, in the context of breast cancer, AI can revolutionize diagnostics and therapeutics by identifying microbial biomarkers associated with subtypes or treatment outcomes.^128^, ^129^ This information can facilitate personalized therapies targeting specific microbial communities, and AI algorithms can predict patients' response to breast cancer treatments, thus minimizing side effects. AI and machine learning (ML) can also identify novel drug targets for breast cancer therapies by analyzing microbiome data, and complex microbial communities can be analyzed to identify key microbial species or functional pathways associated with breast cancer development or treatment response.^130^ Moreover, AI algorithms can be used to design clinical trials that incorporate microbiome data, testing the effectiveness of microbiome‐based interventions alongside standard breast cancer therapies. Thus, AI and ML have the potential to enhance our understanding of the gut microbiome and its role in breast cancer development and progression, enabling the identification of new biomarkers, therapies, and diagnostic tools to improve outcomes for breast cancer patients.

In the context of therapeutic advancements, the microbiota may play a crucial role in predicting individual breast cancer risk and prognosis, as well as the pharmacokinetics, pharmacodynamics, and clinical efficacy of treatments.^41^, ^131^ Recent research has revealed that certain gut commensals, which are present in excess in breast cancer patients compared to healthy individuals, have a negative impact on breast cancer prognosis, are influenced by chemotherapy, and may also affect weight gain and neurological side effects of breast cancer therapies.^132^, ^133^ Clinical researchers are therefore interested in the potential of the gut microbiota as a biomarker for predicting the efficacy of cancer treatment. Studies have shown that the gut microbiota can modulate cancer treatments' efficacy and adverse effects, and that cancer and anticancer therapies interact bidirectionally with the gut microbiota.^134^ In addition, certain gut microbes have been found to protect against inappropriate inflammation and modulate the immune response, which is particularly relevant to breast cancer immunotherapy.^135^ Clinical and preclinical studies have demonstrated that the gut microbiota strongly influences tumor response to immune checkpoint blockade, with follow‐up studies showing an enhancement of the *T* cell response via the activation of antigen presenting cells, such as dendritic cells, in mice models with a stable healthy gut microbiome.^89^, ^136^ However, clinical studies have also shown that dysbiosis of the gut microbiome can induce resistance to immune checkpoint blockade and cause side effects.^137^ The connection between host immunity and the microbiome offers the potential to improve the efficacy of several cancer treatments and provide insights into the causes of treatment‐related side effects. Combining gut microbiota and cancer immunotherapy in innovative ways could result in a more effective personalized medicine approach to cancer treatment soon. Further research is needed to verify these clinical results, and fecal microbial transplantation in murine models is being investigated as a potential way to study the relationship between immune checkpoint blockade and the gut microbiota.^138^

The technological advancements have opened new avenues for studying the gut microbiome's role in breast cancer, but challenges related to data integration, sample collection and processing, and causality versus correlation need to be addressed. One challenge for microbiome‐based therapies is the lack of clear evidence or product standards. The outcomes of these therapies are poorly understood and vary depending on the host and disease, making therapeutic intervention points undefined. Robust clinical trials and validation studies are needed to demonstrate safety, efficacy, consistency, and clinical benefit before wide adoption. However, advancements in systems biology allow for a better understanding of microbiota interactions with the host and its influence on pathophysiology. The microbiome shows promise in diagnostics, prognostication, and therapy. Modulating the microbiota is a way forward for precision oncology. Overcoming these challenges will be crucial for advancing our understanding of the gut microbiome and breast cancer and developing new prevention and treatment strategies.

## 8 COMPUTATIONAL MODELS TOWARD ANALYZING TUMOR‐THERAPY‐MICROBIOME DYNAMICS

Understanding the dynamics of microbial communities and their interactions with host through computational models of varying complexities is an emerging frontier in microbiome research.^127^, ^139^ One such modeling approach pertains to “Genome scale metabolic models (GEM)” integrated with meta‐ omics data which is being widely used by the research community for studying microbe‐microbe and microbe‐host metabolic interactions.^139^ Some of the widely used packages/tools for building and analyzing GEM include “AutoKEGGRec”, “MetaDraft”, “RAVEN Version 2” and so forth.^140^, ^141^ Several studies have discussed the possible “microbiome‐metabolome interactions” occurring in the “tumor microenvironment (TME)” in breast cancer.^142^, ^143^, ^144^, ^145^ For example, two microbial metabolites, lithocholic acid (LCA) and cadaverine, which are synthesized from cholesterol and lysine metabolism respectively, have been suggested to reduce breast cancer cell aggressiveness and proliferation.^140^, ^141^ Few review articles provide comprehensive collation of such mechanistic insights on “breast cancer microbiome‐metabolome interaction” obtained from GEM‐based models.^22^, ^139^ These systems‐level insights form critical building‐elements toward designing improved prognostic and therapeutic techniques for breast cancer.

In addition to “metabolic models”, the “disease (cancer)‐microbiome” crosstalk has been analyzed using “Community‐level models” where the ecological processes governing the structure and dynamics of breast tumor microbiome have been explored.^146^ In the study by Li and colleagues, the application of three modeling approaches, neutral, near‐neutral, and niche‐neutral hybrid models, resulted in novel observations including the effects of malignant tumor on the composition of positively selected bacterial species and microbial dispersal patterns.^146^ Such meta‐community level insights on “tumor‐microbiome dynamics”, obtained through integrated theoretical models, can immensely help in developing more efficient diagnostic and therapeutic regimes.

A third category of in silico modeling approach implemented by the research community toward understanding spatio‐temporal distribution of cells in TME as well as “tumor‐microbiome interaction” pertains to “Agent‐based models (ABM)”.^147^, ^148^ ABMs have been instrumental in qualitative characterization of tumor heterogeneity including (breast) cancer immune response.^148^ For instance, simulation of the ABM constructed by Norton and colleagues provides valuable insights into the effects of breast cancer stem cell proliferation and migration of CCR5+ cancer cells affecting the growth and progression of TNBCs.^147^ Further, ABMs focusing on the intra‐tumor heterogeneity including the “microbiome” component suggest intriguing ways to revert the immune system back into a tumor‐ inhibiting phenotype (from tumor‐enhancing mode), thus improving immunotherapy efficiency in breast cancer treatment.^148^

In summary, multiscale integrated computational models are powerful tools for understanding the complex interplay and spatio‐temporal dynamics of various components of the tumor microenvironment including the “microbiome”. The systems‐level insights obtained from these models, especially those on cancer immunotherapy and immune‐related tumor mechanobiology, hold immense potential in advancing diagnostic, therapeutic and prognostic applications for cancer. Further, novel approaches of combining predictive computational models with AI based models present a set of powerful next‐ gen armories for enabling precision cancer therapy and surveillance.^149^

While the differences in breast oncobiome and their contribution to the disease and treatment response are being explored, understanding racial correlations with microbiome is still in very nascent stages. Parida et. al. report differences in breast oncobiome in White, Black and Asian women.^150^ Given the regional diversity in genetics, diet and environmental exposure, investigations to study the breast oncobiome need to also be conducted in the Indian population. We hence initiated a pilot project exploring the differences in microbiome in ER+ breast cancer patients compared to healthy individuals in the context of menopausal status.

## 9 CURRENT CHALLENGES IN APPLICATION OF MICROBIOME ANALYSIS IN CANCER MANAGEMENT

As evidenced by the growing body of literature, microbiome analysis may prove to be a viable tool in the management of cancers. However, there are several practical challenges that hinder their clinical translation.^42^, ^83^, ^131^ One of the main challenges is the lack of clear evidence or product standards. The mechanisms and outcomes of microbiome‐based therapies are poorly understood and vary depending on the host and the disease. Another challenge is the undefined therapeutic intervention points for microbiome‐based therapies. Robust clinical trials and validation studies are required to demonstrate the safety, efficacy, consistency, and clinical benefit of microbiome‐based therapies before they can be widely adopted. Despite the aforementioned challenges, the current advancements in systems biology now enable a deeper understanding of the complex interactions of the microbiota with the host and its influence on pathophysiology. The microbiome is thus a promising new avenue for not only diagnostics and prognostication but also therapy in breast cancer context.

## 10 OUR EXPERIENCES FROM THE ESTROGEN AND MICROBIOME IN BREAST‐CANCER EPIDEMIOLOGY AND RISK (EMBER) STUDY

**FIGURE 2:**
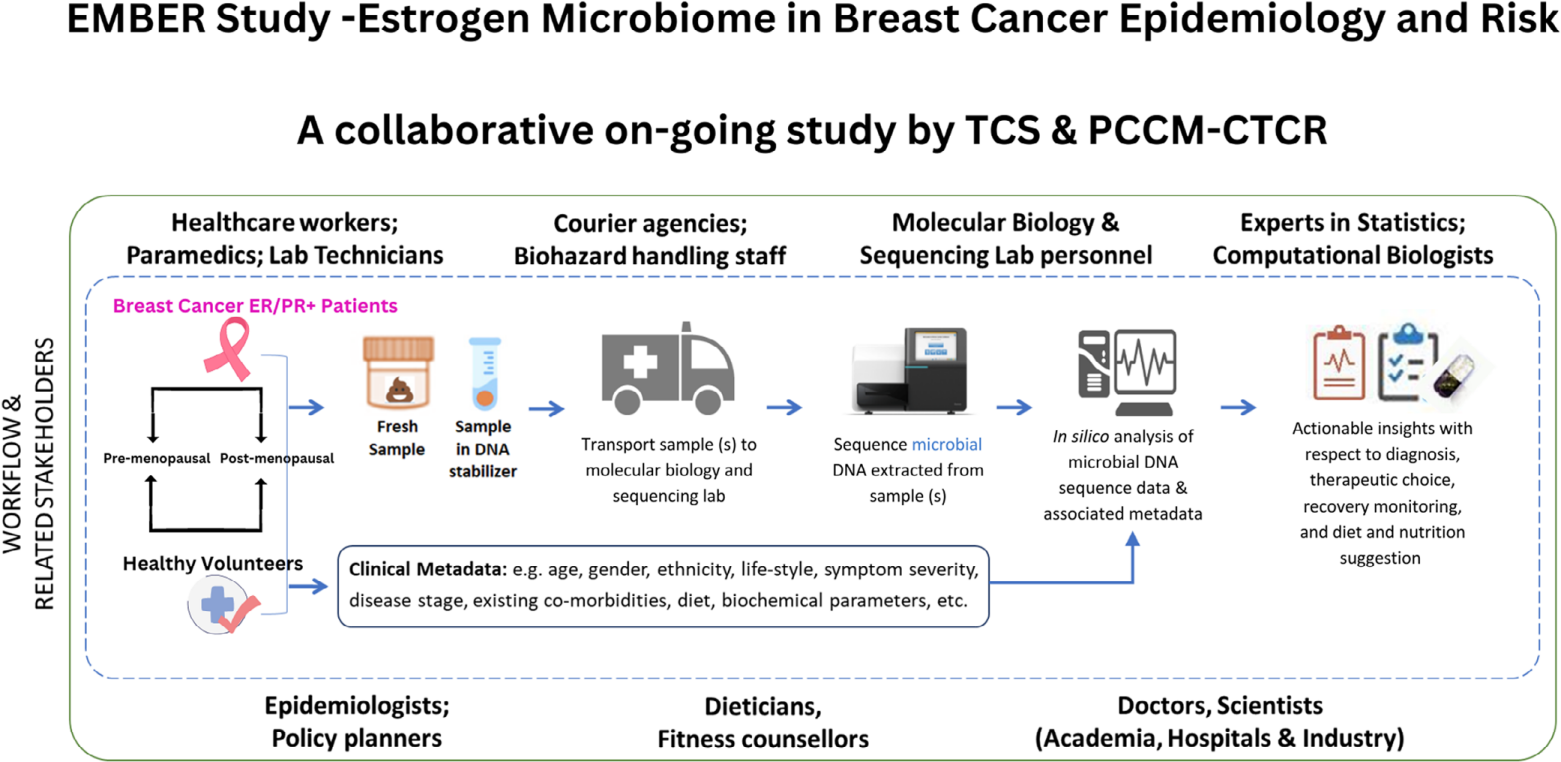
A schematic diagram indicating the EMBER Study (2020–2022) workflow that was followed for microbiome sample collection, sequencing, and computational analysis. Various stakeholders who are involved (or have active interest) in one/more stages of the entire workflow are also depicted.

An ongoing collaborative pilot study, EMBER, at our center focuses on ER/PR+ Breast Cancer to investigate the correlation between ER/PR+ breast oncobiome and clinicopathological parameters. The study is aimed to analyze the gut and salivary microbiomes of ER/PR+ breast cancer patients, in order to gain insights into multiple aspects of the microbiome. Insights obtained in this study could potentially give a clue for microbiome as “early” surrogate's markers for assessing the risk of breast cancer in the Indian context. At present, the study is in the data analysis phase and Figure 2 depicts the workflow of the study. Our study design included recruitment of pre‐ and post‐menopausal healthy individuals and breast cancer patients in equal numbers, to identify the differential microbiome signatures. Sample collection included blood, saliva, and stool sample from newly diagnosed breast cancer patients and healthy individuals. However, we observed a few limitations in our study. First, in our cohort frequency of ER/PR‐positive pre‐menopausal patients was much lower compared to post‐menopausal patients. Workflow issues were observed more in the breast cancer arm as compared to the healthy cohort and can be attributed to patient psychology in the face of recent breast cancer diagnosis. Also, patient fall‐out rate was observed to be affected by various factors, for example, the collection of biological specimens, especially stool samples. In this particular study we also faced issues in recruitment due to the pandemic. In phase 2 of our study, we plan to take appropriate measures to mitigate the aforementioned limitations. Moreover, since our ongoing study is limited only to the ER/PR subtype, we suggest that future oncobiome studies should include all molecular subtypes of breast cancer. However, based on our experience, we believe that further research is needed to validate the findings from our study across multiple centers in India.

**FIGURE 3:**
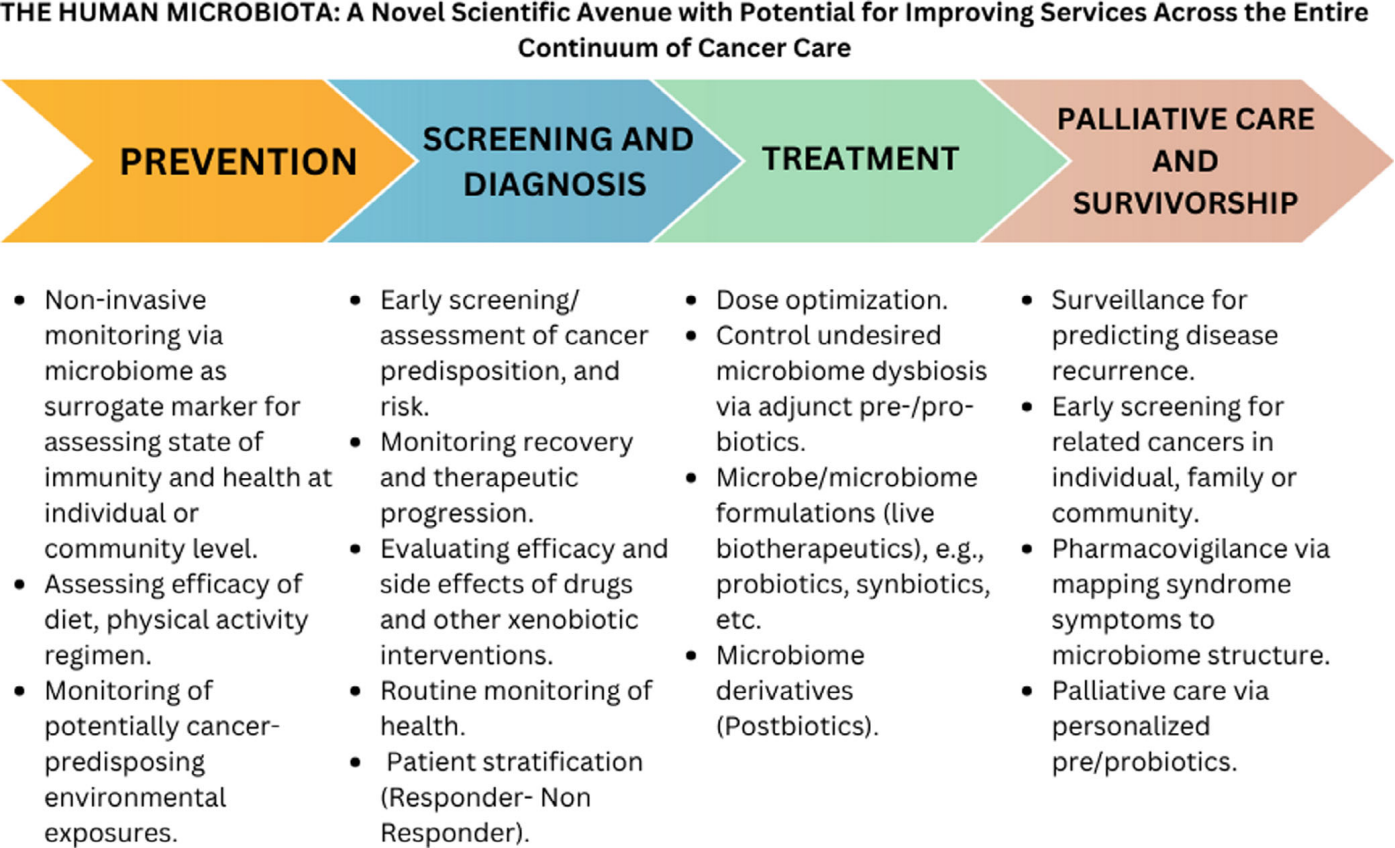
The Human Microbiota: A Novel Scientific Avenue with Potential for Improving Services Across the Entire Continuum of Cancer Care. The figure illustrates the potential role of the human microbiota in various stages of cancer care, including prevention, screening, diagnosis, treatment, palliative & supportive care.

The World Health Organization (WHO) espouses systematic strengthening of health systems in every nation (irrespective of its economic or resource constraints) with the twin objective of curing disease and elongating life. The “human microbiota” has recently emerged as one such “novel” scientific avenue exhibiting immense potential with respect to revolutionizing and re‐defining the way(s) by which several diseases including cancer, can be diagnosed, monitored, treated and managed. In fact, the WHO's World Cancer Report–2020 has a dedicated sub‐section (3.10) enlisting human microbiota as one of key biological processes implicated in cancer development.^151^ In Figure 3 we provide a schematic representation of the scope of human microbiota with respect to cancer care.

## 11 CONCLUSION

In the recent update to Hallmarks of Cancer, Polymorphic microbiomes have been added as a hallmark. Several studies suggest that microbiomes influence the immunomodulation and genomic instability, but whether other hallmarks of cancer can also be influenced by the microbiota is yet to be determined. Modulation of microbial communities that might influence tumorigenesis and treatment response is an emerging modality for breast cancer management. However as elaborated in the review, there are still many challenges in the clinical application of these microbiome‐based therapies. These are further compounded by population‐specific changes in microbiome composition. To the best of our knowledge there are no published studies from the Indian population on the differential microbiome composition in breast cancer cases. Thus, although microbiome modulation is the way forward in cancer management, more population‐specific studies dissecting the role and mechanism of microbiome influence on breast cancer progression are the need of the hour.^42^, ^83^, ^131^, ^152^

## AUTHOR CONTRIBUTIONS

**Asmita Jotshi:** Data curation (equal); methodology (equal); visualization (equal); writing – original draft (equal); writing – review and editing (equal). **Krishna Kishore Sukla:** Data curation (equal); methodology (equal); validation (equal); visualization (equal); writing – original draft (equal); writing – review and editing (equal). **Mohammed Monzoorul Haque:** Data curation (equal); project administration (equal); resources (equal); visualization (equal); writing – original draft (equal); writing – review and editing (equal). **Chandrani Bose:** Conceptualization (equal); data curation (equal); investigation (equal); visualization (equal); writing – original draft (equal); writing – review and editing (equal). **Binuja Varma:** Conceptualization (equal); data curation (equal); formal analysis (equal); investigation (equal); project administration (equal); supervision (equal); visualization (equal); writing – original draft (equal); writing – review and editing (equal). **Chaitanyanand B Koppiker:** Conceptualization (supporting); data curation (supporting); funding acquisition (lead); investigation (supporting); methodology (supporting); project administration (lead); resources (lead); supervision (lead); validation (supporting); visualization (supporting); writing – original draft (supporting); writing – review and editing (supporting). **Sneha Joshi:** Conceptualization (lead); data curation (lead); formal analysis (lead); funding acquisition (supporting); investigation (lead); project administration (lead); resources (lead); supervision (lead); visualization (lead); writing – original draft (lead); writing – review and editing (lead). **Rupa Mishra:** Conceptualization (lead); data curation (lead); formal analysis (lead); funding acquisition (supporting); investigation (lead); project administration (lead); resources (lead); supervision (lead); visualization (lead); writing – original draft (lead); writing – review and editing (lead).

## CONFLICT OF INTEREST STATEMENT

The authors do not have any conflict of interest.

## ETHICS STATEMENT

The EMBER Study approval was granted by the Independent ethics committee of Prashanti Cancer Care Mission. (DCGI/CDSCO Registration Number: ECR/298/Indt/MH/2018 (dated May 14 2018).

## Data Availability

Data availability statement is not applicable.
